# RIPK4 Downregulation Reduces ABCG2 Expression, Increasing BRAF-Mutated Melanoma Cell Susceptibility to Cisplatin- and Doxorubicin-Induced Apoptosis

**DOI:** 10.3390/biom14121573

**Published:** 2024-12-10

**Authors:** Bartlomiej Olajossy, Norbert Wronski, Ewelina Madej, Joanna Komperda, Małgorzata Szczygieł, Agnieszka Wolnicka-Glubisz

**Affiliations:** 1 Department of Biophysics and Cancer Biology, Faculty of Biochemistry, Biophysics and Biotechnology, Jagiellonian University, Gronostajowa Street 7, 30-387 Krakow, Poland; bartek.olajossy@doctoral.uj.edu.pl (B.O.); norbert.wronski@doctoral.uj.edu.pl (N.W.); em939@cam.ac.uk (E.M.); gosia.szczygiel@uj.edu.pl (M.S.); 2 Doctoral School of Exact and Natural Sciences, Jagiellonian University, 30-387 Krakow, Poland

**Keywords:** MPR, melanoma, RIPK4, cyclosporin A, cisplatin, doxorubicin

## Abstract

Melanoma cells remain resistant to chemotherapy with cisplatin (CisPt) and doxorubicin (DOX). The abnormal expression of Receptor-Interacting Protein Kinase 4 (RIPK4) in certain melanomas contributes to tumour growth through the NFκB and Wnt/β-catenin signalling pathways, which are known to regulate chemoresistance and recurrence. Despite this, the role of RIPK4 in response to chemotherapeutics in melanoma has not been reported. In this study, we examined how the downregulation and overexpression of RIPK4 affect the sensitivity of BRAF-mutated melanoma cells (A375 and WM266.4) to CisPt and DOX along with determining the underlying mechanism. Using two RIPK4 silencing methods (siRNA and CRISPR/Cas9) and overexpression (dCas9-VPR), we assessed CisPt and DOX-induced apoptosis using caspase 3/7 activity, annexin V/7AAD staining, and FASC analysis. In addition, qRT-PCR and Western blotting were used to detect apoptosis-related genes and proteins such as cleaved PARP, p53, and cyclin D1. We demonstrated that the overexpression of RIPK4 inhibits, while its downregulation enhances, CisPt- or DOX-induced apoptosis in melanoma cells. The effects of downregulation are similar to those observed with pre-incubation with cyclosporin A, an ABCG2 inhibitor. Additionally, our findings provide preliminary evidence of crosstalk between RIPK4, BIRC3, and ABCG2. The results of these studies suggest the involvement of RIPK4 in the observed resistance to CisPt or DOX.

## 1. Introduction

Chemotherapy based on platinum is widely used in the treatment of cancers [1]. By the early 2000s, the global sales of platinum-based anticancer drugs had reached 2 billion USD, with approximately 50% of patients undergoing treatment with cisplatin (CisPt), an inorganic compound containing platinum [2,3,4]. Cisplatin, a potent first-generation platinum-based anticancer drug, effectively treats various tumours, including lung, esophageal, breast, gastric, lymphoma, ovarian, head, and neck cancers [5,6,7]. Acting as a cell-cycle non-specific drug, it targets DNA and induces cell death by accumulating reactive oxygen species (ROS) in mitochondria, triggering oxidative stress-mediated cell death [8,9,10]. In addition, treatment of CisPt has been carried out with varying success due to side effects and drug resistance, which depends on a number of factors, such as reduced drug accumulation, inactivation of the drug by binding to various proteins, increased DNA repair, and alteration of various apoptosis signalling proteins [11,12,13,14].

Although melanoma cells remain resistant to CisPt-based chemotherapy, CisPt has been widely investigated in combination therapies due to its in vivo immunomodulatory properties, which may enhance the efficacy of immunotherapy, as demonstrated in the treatment of glioma and urothelial cancer [15,16,17].

Melanoma is characterised by a high tumour mutation burden and heterogeneity. Based on the pattern of the most prevalent significantly mutated genes, it is classified into four genomic subtypes: mutated BRAF, mutated RAS, mutated NF1, and Triple-WT (wild type), wherein the enrichment of KIT mutations is characteristic of the wild type. Among these mutations, BRAF and NRAS are two of the most common, but also mutually exclusive mutated oncogenes recognised in melanoma [18,19]. Mutations in the BRAF gene occur in ~40–85% of melanoma cases, with the lowest frequency in primary melanomas, increased in metastatic sites, and the highest in recurrent melanomas, while NRAS mutation occurs in ~25% [19,20].

The therapeutic advancements achieved with BRAFV600/MEK inhibitors and immune checkpoint inhibitors in melanoma remain unsatisfactory. Despite the immune response, the mortality rate in patients with stage IV melanoma remains high. Moreover, the toxicity of both current and new immunotherapy combinations remains a problem that needs further investigation [21,22].

Receptor-interacting protein kinase (RIPK4) is a highly conserved member of the RIP family of serine/threonine kinases whose function is crucial in integrating various stress signals [23]. Based on numerous studies, it is evident that RIPK4 is an important mediator in NFκB activation and Wnt signalling maintaining epithelial homeostasis [24,25,26]. Moreover, disruption in its expression has been associated with the tumorigenesis of several malignancies [27,28]. Depending on the cellular context, RIPK4 can act either as a tumour suppressor or a tumour promoter [24,29,30].

Our previous studies strongly indicate that RIPK4 plays an oncogenic role in melanoma [24,25]. Global analysis of gene expression changes upon RIPK4 silencing reveals a complex role for this kinase in regulating adhesion, migration, proliferation, and inflammatory processes in melanoma cells [31]. We showed that RIPK4 is involved in signal transduction through the NFκB [24] and Wnt/β-catenin pathways, regulating the Wnt3A-induced invasive potential of melanoma cells and the growth of xenografts in mice [25]. In contrast to pancreatic cancer [27], melanoma RIPK4 appears not to be a component of the BRAF/MEK/ERK cascade signalling pathway. Nevertheless, its expression is downregulated following treatment with BRAF inhibitors, such as dabrafenib and vemurafenib [30]. Wang et al. showed that the downregulation of RIPK4 enhances the sensitivity of Tca-8113 cells to cisplatin in squamous cell carcinoma of the tongue [32]. Additionally, it has been identified that YTHDF1, in a METTL3-dependent manner, triggers the m6A modification of *RIPK4*, subsequently suppressing the degradation of its mRNA. This mechanism is associated with drug resistance of epithelial ovarian cancer [33]. Silencing of RIPK4 through siRNA or knockout via the CRISPR-Cas9 system does not induce apoptosis in melanoma cells. However, it may reduce the proliferative potential of both in vitro cells and in vivo mouse models of tumour growth [25,30,31]. Therefore, we hypothesised that altering RIPK4 expression impairs NFκB and Wnt/β-catenin pathways, also known to regulate chemoresistance and recurrence and increase the sensitivity of melanoma cells to CisPt treatment.

To explore the molecular mechanism underlying the role of RIPK4 in cell resistance to CisPt, we modulated the expression of this protein by applying siRNA for transient silencing and CRISPR/Cas9 for both the knockout and overexpression of RIPK4 in WM266.4 and A375 cell lines. Using a combination of doxorubicin (DOX) and cyclosporine (CsA)—an inhibitor of the ABCG2 transporter responsible for DOX resistance [34]—we evaluate the role of RIPK4 in cisplatin-induced cell death. Our findings confirm that RIPK4 can regulate resistance to CisPt and DOX, suggesting its potential as a target in a multi-target chemotherapy-based strategy.

## 2. Materials and Methods

### 2.1. Cell Culture and Treatment

In the study, human melanoma cell lines BLM (wild type for BRAF, NRAS mutated) and mutated BRAF (SKMEL-28, WM266.4, and A375) were used. WM266.4 and A375 cells were co-transduced with lentiviral vectors encoding Cas9 and sgRNA targeting the RIPK4 gene or a negative control. After selection with puromycin and blasticidin, clonal cell lines were established, and RIPK4 knock-out was confirmed using Western blotting and Sanger sequencing. For further details, refer to our previous work [25]. BLM and WM266.4 were supplied by the Department of Biophysics and Cancer Biology, Jagiellonian University, SKMEL-28 was a gift from Dr. Konrad Kleszczynski, University of Munster, Germany, and A375 was obtained from ATCC. Cells were cultured in RPMI1640 medium supplemented with 10% FBS and antibiotics (penicillin 150 U/mL, streptomycin 100 µg/mL) as previously described [24,25,30]. The cell cultures were kept at 37 °C at 5% CO_2_ and 95% humidity. Doxorubicin (4 mM) and cyclosporine (5 mM) were dissolved in DMSO, aliquoted, and stored at −20 °C. Cisplatin (20 mM) was prepared in DMSO freshly prior to experiments. The drugs were added to the cell cultures for 6 to 24 h. CsA was added 1 h before the treating cells with the drugs. The concentration of DMSO does not exceed 0.05%. All experiments were performed with mycoplasma-free cells. The list of general chemicals and reagents is shown in Supplementary Materials, Table S1.

### 2.2. Transfection with Small Interfering RNA (siRNA)

WM266.4 cells were seeded at density 3 × 10^5^ on 35 mm cell culture dishes 24 h before the transfection. Transfection was performed as previously described [24,30] using RIPK4-specific Silencer Select or Silencer Select Negative Control, in the presence of Lipofectamine 2000, according to manufacturer’s recommendations. The ability of siRNA to inhibit *RIPK4* gene expression was confirmed using Western blot analysis.

### 2.3. Overexpression of RIPK4

A375 cells with stable Cas9 expression were obtained as described [30] and seeded at a density of 5 × 10^4^ in 35 mm cells per well cell culture plates 24 h before transfection. Cells were transfected with two different synthetic sgRNAs (crRNA: tracrRNA targeting the RIPK4 gene) or with a non-targeting control (crRNA) using DharmaFECT 4 transfection reagent according to the manufacturer’s protocol. RIPK4 expression levels in A375-dCas9-VPR-crRNA transfected cells were verified 72 h after transfection using Western blot analysis.

### 2.4. MTT

The cell viability of negative control cells and cells with diminishing RIPK4 was analysed using MTT. MTT was added for 1 h to the final concentration of 500 ng/mL. After removal of the medium, the formazan crystals were dissolved in DMSO: ethanol (1:1, *v*/*v*). The absorbance was measured on a microplate reader (Tecan Group Ltd. Mannedorf, Switzerland) at 570 nm.

### 2.5. Apoptosis Assays

Caspase activity 3/7 was determined using the luminescent Caspase-Glo-3/7 Assay Kit (Promega, Madison, WI, USA) according to the manufacturer’s protocol using a multimode microplate reader Synergy H1 (BioTek, Winooski, VT, USA). Briefly, equal amounts of cell lysates (4 μg of proteins) were filled in white 96-well plates with RIPA buffer to the final volume of 40 μL. Next, 40 μL of Caspase-Glo 3/7 Reagent was added and shaken for 2 min. Then, the plates were kept in the dark at room temperature for 1.5 h.

Annexin V and 7-amino-actinomycin D (7AAD) staining was performed as previously described [35]. Cells were collected 24 h after the treatment, washed with Hepes buffer, and incubated with Annexin V-FITC in the dark for 10 min. Subsequently, cells were washed with Hepes buffer, resuspended, and stained with 5 μL 7-AAD for an additional 5 min. 1 × 10^4^ cells were collected with the FACSCalibur instrument (BD Biosciences, San Jose, CA, USA) and analysed using the CellQuest software (BD Biosciences, San Jose, CA, USA). The measurement was carried out using 488 nm excitation, a 510–570 nm band-pass emission filter for the detection of fluorescein isothiocyanate (FITC), and a 650 nm long-pass emission filter for 7-AAD detection.

### 2.6. Quantitative Real-Time PCR

Total RNA was isolated using Total RNA Mini Plus according to the manufacturer’s protocol. qRT-PCR was performed using a thermal cycler qTOWER3 (Analytik Jena, Jena, Germany) as previously described [24]. All TaqMan primers purchased from Thermo Fisher Scientific/Invitrogen are included in Table S2 (Supplementary Materials). The relative levels of the transcripts were quantified using the ΔΔCT method, with GAPDH as a reference gene.

### 2.7. Western Blot

Cells were lysed in RIPA buffer supplemented with Complete Protease Inhibitor Cocktail and PhosSTOP Phosphatase Inhibitor Cocktail. 20 µg of total protein was separated on 12% Bis-Tris gels (TGX™ FastCast™ Acrylamide Kit, BioRad, Hercules, CA, USA). Following the electrophoresis and transfer, PVDF membranes (Millipore, Billerica, MA, USA) were blocked for 40 min with 4% bovine serum albumin in TBS-T buffer and incubated with primary antibody at 4 °C overnight. The blots were cut prior to hybridisation with antibodies. The list of used antibodies is provided in Supplementary Materials, Table S3. After three washes, the incubation with secondary antibody (1 h at room temperature), and an additional three washes, the detection was performed using Clarity Western ECL Substrate with the ChemiDoc (Bio-Rad, Hercules, CA, USA) detector and ImageLab 5.2.1. software.

### 2.8. Statistical Analysis

All data were analysed using two-way ANOVA or a two-tailed unpaired Student *t-test* using the GraphPad Prism software (version 9.0.0, GraphPad Software, La Jolla, CA, USA). The number of independent experiments and replicates for each experiment is indicated in the figure legends. *p* < 0.05 was considered significant and labelled by an asterisk in the figures unless otherwise indicated in figure legends.

## 3. Results

### 3.1. RIPK4 Downregulation Enhances Sensitivity of Melanoma Cells Towards CisPt Treatment

**Figure 1:**
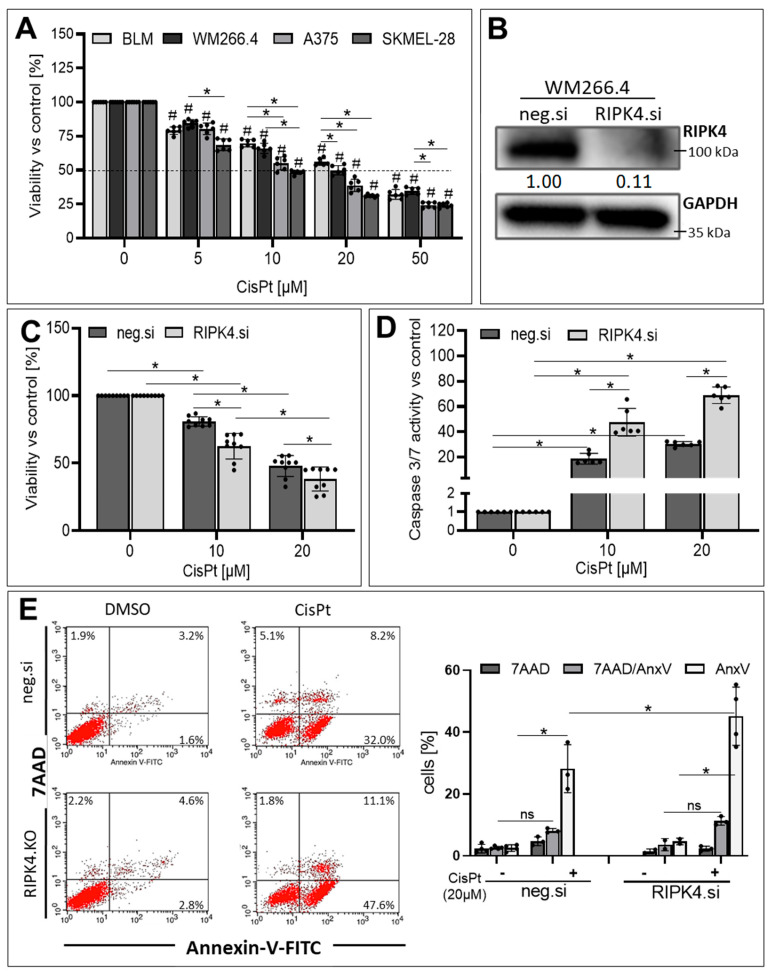
The effect of RIPK4 silencing on CisPt-induced apoptosis of melanoma cells: (**A**) Viability of melanoma (BLM, WM266.4, A375, SKMEL-28) treated with cisplatin (CisPt) at 24 h using the MTT assay. (**B**) Efficiency of RIPK4 silencing in WM266.4 cells transfected with RIPK4.si1 RNA, or neg.si RNA was analysed after 48 h using Western blotting. (**C**–**E**) CisPt decreases WM266.4 viability and induced apoptosis 24 h after the treatment in both neg.si and RIPK4.si transfected cells. (**C**) MTT; *n* = 3, (**D**) caspase3/7 activity; *n* = 3 and (**E**) Annexin-V-FITC (AnxV)/7AAD double staining and FASC analysis. Dead cells (necrotic: 7AAD^+^, early apoptotic: AnxV^+/^7AAD^−^ and late apoptotic: AnxV^+^/7AAD^+^); *n* = 3. Each bar represents the mean ± SD of three to four biological replicates. ns—not significant, # indicates *p* < 0.05 vs. control (untreated cells), * indicates *p* < 0.05 between the probes as marked by the line. Original images can be found in Figure S1.

Our previous data indicate that various melanoma cell lines exhibit a wide range of RIPK4 expression levels, ranging from high (WM266.4, SKMEL-28), medium (A375) to low (BLM), as described [25]. A significant decrease in cell viability was observed in all cell models after treatment with CisPt, ranging from 30% to 50% for 20 µM (Figure 1A). Therefore, this concentration was chosen for further study.

**Figure 2:**
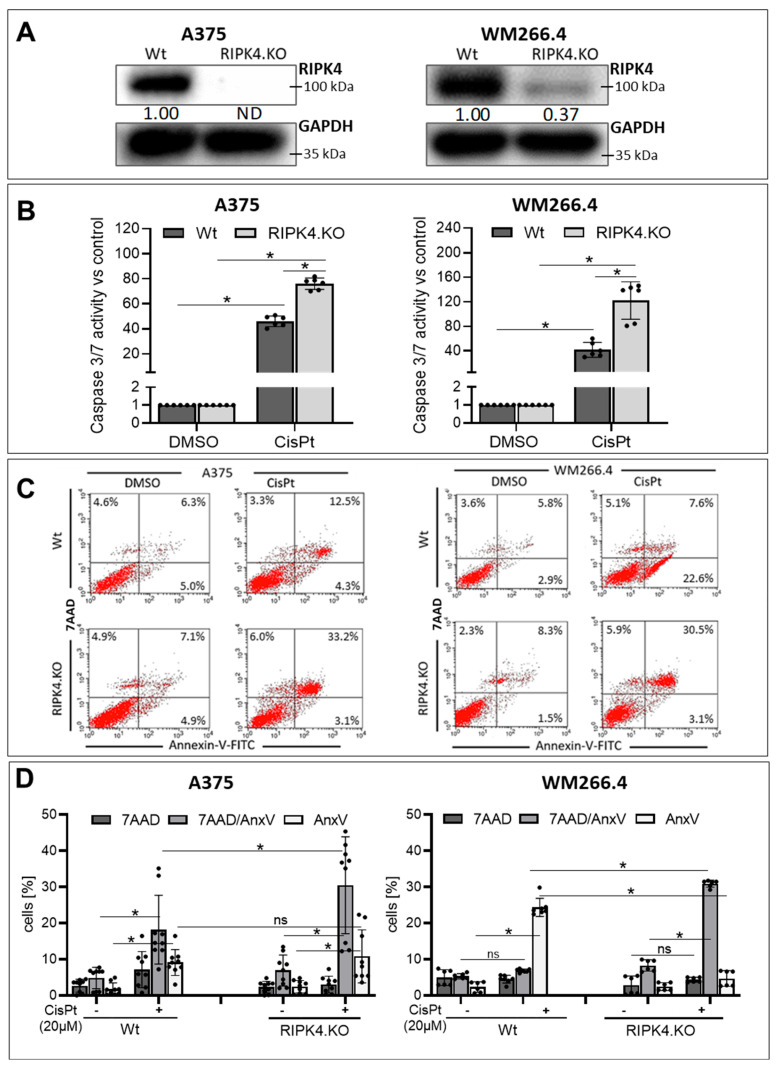
Stable downregulation of RIPK4 enhances apoptosis induced by CisPt-induced apoptosis in A375 and WM266.4 melanoma cells 24 h after the treatment: (**A**) The level of RIPK4 in A375^RIPK4.KO^, WM266.4^RIPK4.KO^cells, and their parental lines (wild-type, Wt). (**B**) Caspase3/7 activity, *n* = 3. (**C**) Annexin V-FITC (AnxV)/7AAD double staining and FASC analysis. (**D**) Dead cells (necrotic: 7AAD^+^, early apoptotic: AnxV^+^/7AAD^−^, and late apoptotic: AnxV^+^/7AAD^+^). Each bar represents the mean ± SD of three biological replicates. * *p* < 0.05 were considered significant; ns—not significant. Original images can be found in Figure S2.

Surprisingly, among the cell lines, WM266.4 cells, which exhibited the highest levels of RIPK4, as well as BLM cells with the lowest levels, were the most resistant to CisPt treatment. To investigate the role of RIPK4 in CisPt-induced cell death, we selected the WM266.4 cell line for silencing RIPK4 expression using siRNA, the effectiveness of which was confirmed using Western blotting (Figure 1B). The apoptosis of cells treated with CisPt was evaluated by assessing the activation of caspase 3/7 and staining with Annexin V/7AAD, which allow to discriminate early apoptosis (AnxV^+^/7AAD^−^) from late apoptosis (AnxV^+^/7AAD^+^) and necrosis (AnxV^−^/7AAD^+^). As expected, RIPK4.si transfected cells were more sensitive to CisPt-induced apoptosis (20 µM) than neg.si transfected cells (Figure 1C–E), as manifested by a higher increase in caspase 3/7 activity (70-fold for RIPK4. si vs. 30 for control) and a higher percentage of apoptotic cells of 58% (for the sum of AnxV ^+^/7AAD^+^ and AnxV^+^/7AAD^−^ cell populations) compared to 40% in control. As we observed the effect of transient RIPK4 silencing in WM266.4 cells, we extended our studies to examine whether the stable downregulation of RIPK4 has a similar effect in sensitising cells to CisPt. For this purpose, the gene encoding RIPK4 was silenced in two melanoma cell lines A375 and WM266.4 cells using the CRISPR/Cas9 technique [25]. Figure 2 illustrates that stable knockout of RIPK4 increases CisPt-induced apoptosis in A375 and WM266.4 melanoma cells (Figure 2A–D).

The caspase 3/7 activity in A375^RIPK4.KO^ and WM266.4^RIPK4.KO^ cells increase significantly compared to their wild-type (Wt) parental lines (approximately from 60- to 80-fold for A375 and from 50- to 150-fold for WM266.4) (Figure 2B). Interestingly, Annexin V/7AAD staining revealed that A375 and WM266.4 cells differ in their response to CisPt. In A375 cells, late apoptosis predominated (12.5% AnxV^+^/7AAD^+^), while early apoptosis was less frequent (4.3% AnxV^+^/7AAD^−^). In contrast, WM266.4 cells exhibited mainly early apoptosis (22.6% AnxV^+^/7AAD^−^) following CisPt treatment. RIPK4 knockout sensitised A375 and WM266.4 cells to CisPt-induced apoptosis, although in different manners. In A375 cells, RIPK4 knockout increased the late apoptotic cell population from 12.5% in wild-type (Wt) cells to 33.2% in A375 ^RIPK4.KO^ cells. Conversely, in WM266.4 cells, 22.6% of Wt cells were identified as early apoptotic and 7.6% as late apoptotic cells; meanwhile, in WM266.4^RIPK4.KO^ cells, 3.1% were early apoptotic and 30.5% were late apoptotic cells, indicating a more efficient induction of apoptosis in WM266.4^RIPK4.KO^ cells within a shorter time frame.

**Figure 3:**
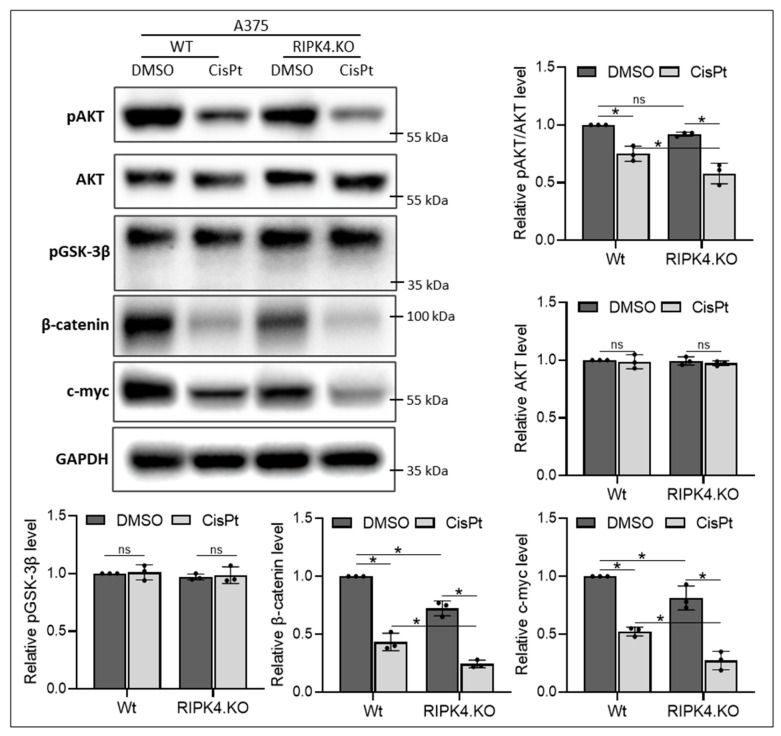
CisPt and RIPK4.KO attenuates Wnt/β-catenin signalling in A375 melanoma cells 24 h after treatment: The protein levels of pAKT, AKT, pGSK3β, β-catenin, c-myc in knockout cells (RIPK4.KO) and their parental lines (Wt) were assessed using Western blotting with densitometry, *n* = 3. GAPDH servers as loading control. Each bar represents the mean ± SD of three biological replicates. * *p* < 0.05 was considered significant. ns—not significant. Original images can be found in Figure S3.

**Figure 4:**
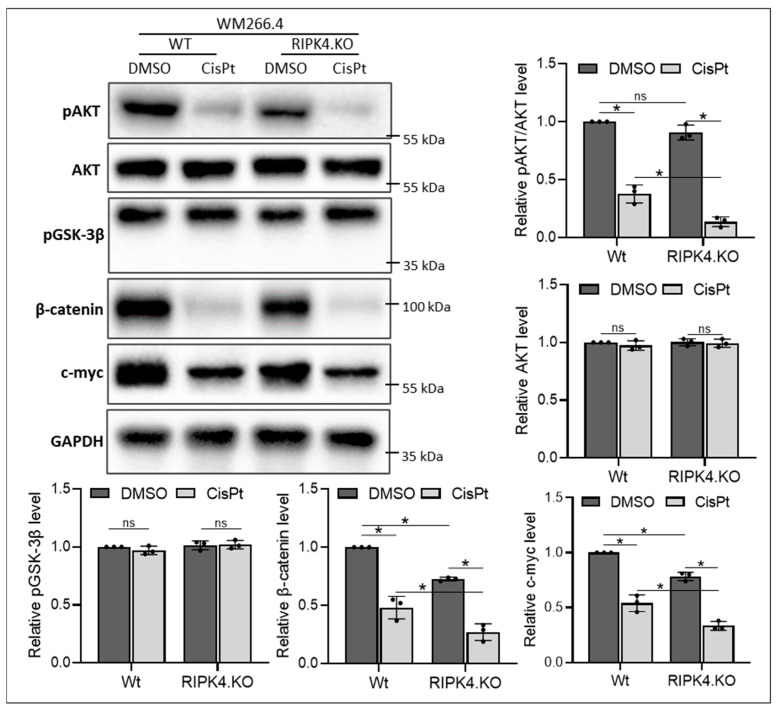
CisPt and RIPK4.KO attenuates Wnt/β-catenin signalling in WM266.4 melanoma cells 24 h after treatment. The protein levels of pAKT, AKT, pGSK-3β, β-catenin, and c-myc in knockout cells (RIPK4.KO) and their parental lines (Wt) were assessed using Western blotting with densitometry, *n* = 3. GAPDH servers as loading control. Each bar represents the mean ± SD of three biological replicates. * *p* < 0.05 was considered significant. ns—not significant. Original images can be found in Figure S4.

As our previous studies demonstrated Wnt/β-catenin signalling in the mechanism of action of RIPK4, in this study, we investigated the association of these pathways with RIPK4 and CisPt. We observed that both CisPt and the RIPK4 knockout alone, while having no effect on GSK3β activity, decrease AKT activity and downregulate β-catenin and c-myc, a target gene (Figure 3 and Figure 4). Furthermore, this effect is enhanced in RIPK4.KO cells treated with CisPt.

### 3.2. RIPK4 Knockout Regulates the Levels of Apoptosis-Related Genes and the ABCG2 Protein in Melanoma Cells

**Figure 5:**
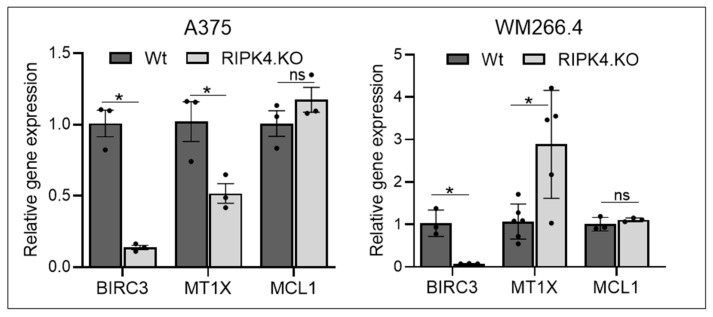
The effect of CRISPR/Cas9 mediated RIPK4 downregulation in A375 (right) and WM266.4 (left) cells on the expression of pro- and anti-apoptotic genes: Transcript levels of BIRC3, MCL-1, MT1X, and in A375^RIPK4.KO^, WM266.4^RIPK4.KO^cells and their parental lines normalised to GAPDH; *n* = 3. Each bar represents the mean ± SD of three to six biological replicates. * *p* < 0.05 were considered significant; ns—not significant.

To decipher the mechanisms of RIPK4-related CisPt resistance, we examined the expression of anti-apoptotic gene myeloid cell leukemia-1 (MCL1), baculoviral IAP repeat containing 3 (BIRC3) that encodes the protein cellular inhibitor of apoptosis protein 2, and metallothionein (MT1X), a protein involved in metal ion homeostasis, DNA damage, oxidative stress, and carcinogenesis. Figure 5 shows that the RIPK4 knockout has distinct effects on MT1X expression. A375^RIPK4.KO^ cells exhibit a reduced level of MT1X expression (mean = 0.52; *p* = 0.03) compared to wild-type cells, in contrast to WM266.4^RIPK4.KO^ cells (mean = 2.63; *p* = 0.01).

**Figure 6:**
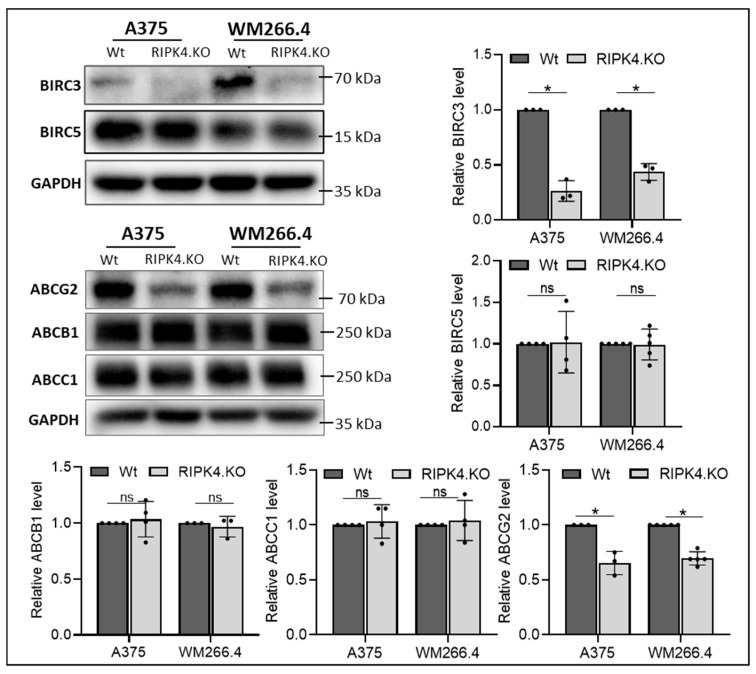
The effect of CRISPR/Cas9 mediated RIPK4 downregulation in A375 and WM266.4 cells on BIRC3,-5 and MPRs proteins. The protein levels of BIRC5, BIRC3, ABCB1, ABCC1, and ABCG2 in knockout cells (RIPK4.KO) and their parental lines (Wt) were assessed using Western blotting with densitometry, *n* = 3. GAPDH servers as loading control. Each bar represents the mean ± SD of three to five biological replicates. * *p* < 0.05 was considered significant. ns—not significant. Original images can be found in Figure S5.

No differences in MCL1 expression were observed between wild type and RIPK4.KO cells. Furthermore, our previous study showed a siRIPK4-mediated reduction in BIRC3 expression in WM266.4 cells [31], also confirmed in the present study in A375^RIPK4.KO^ and WM266.4^RIPK4.KO.^ cells at mRNA and protein levels (Figure 4 and Figure 5). Considering the positive correlation between BIRC5 overexpression and resistance to chemotherapy, contributing to apoptosis avoidance by blocking apoptosis signalling pathways [36], we tested levels of the BIRC5 in knockout cells. However, our analysis did not reveal any differences in BIRC5 at the protein level (Figure 6). Furthermore, we investigated the levels of multidrug resistance proteins (MPR) such as ABCB1, ABCC1, and ABCG2. As shown in Figure 6, the deletion of RIPK4 significantly reduces only ABCG2, circa 65% for A375^RIPK4.KO^ and 70% for WM266.4^RIPK4.KO^ at the protein level.

### 3.3. Downregulation of RIPK4 Enhanced the Sensitivity of Melanoma Cells to CisPt-Induced Apoptosis, Similar to Cyclosporin A

**Figure 7:**
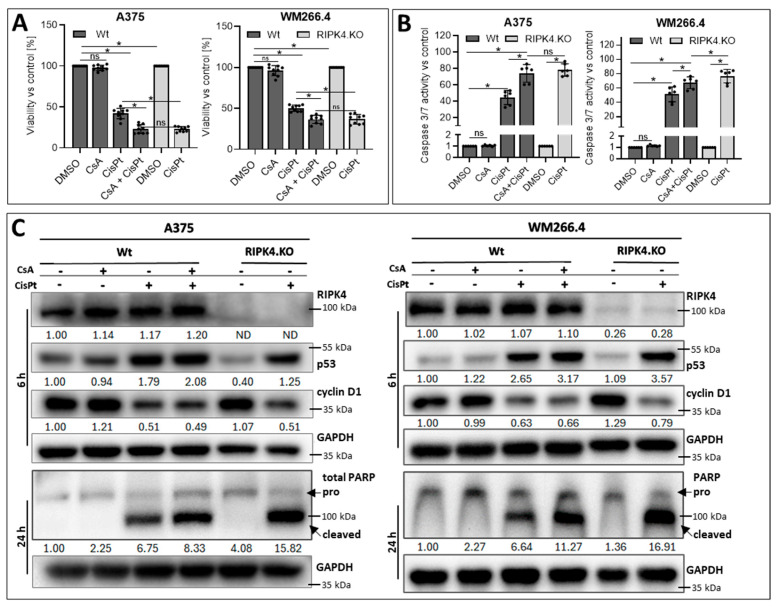
Effects of cyclosporin A and CRISPR/Cas9 mediated RIPK4 downregulation on CisPt-induced apoptosis of melanoma cells 24 h after the treatment: (**A**) Viability by MTT assay; *n* = 3. (**B**) caspase3/7 activity; *n* = 3. Each bar represents the mean ± SD of three biological replicates in duplicates. * *p* < 0.05 were considered significant. (**C**) Protein levels of RIPK4, p53, cyclinD1, and total Poly (ADP-ribose) polymerase (PARP) after indicated time periods using Western blotting. GAPDH was used as a loading control. ns—not significant. Original images can be found in Figure S6.

Since the ABCG2 protein level is significantly decreased in RIPK4 knockout cells, we investigated whether the effect of RIPK4 knockout on sensitivity to CisPt is comparable to that of incubating cells with CsA. Wild-type cells incubated with CsA (5 µM) for 1 h showed no effect on cell viability (Figure 7).

**Figure 8:**
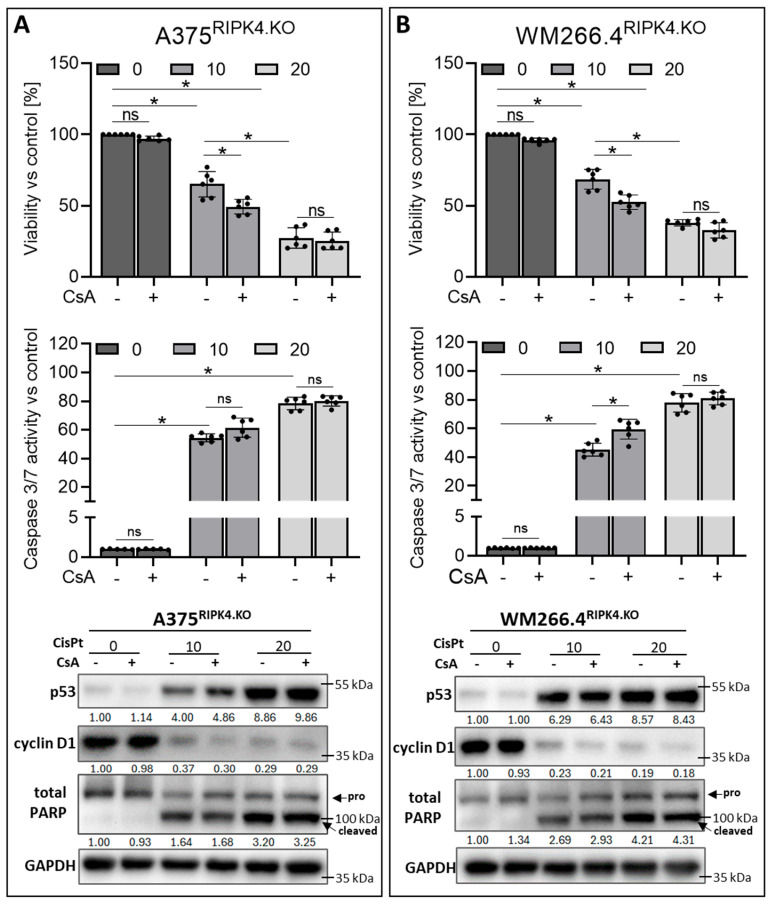
RIPK4 knockout enhances the pro-apoptotic effect of CisPt pre-stimulated cells with CsA: (**A**) A375^RIPK4.KO^, (**B**) WM266.4^RIPK4.KO^ were pre-incubated for 1 h with CsA (5 μM) and CisPt (10 and 20 μM), or DMSO, as a control for 24 h. The cells viability by MTT assay, *n* = 3 (upper panel), caspase 3/7 activity (middle panel), *n* = 3. Each bar represents the mean ± SD of three biological replicates in duplicate. * *p* < 0.05 were considered significant. Protein levels of p53, cyclin D1, and total Poly (ADP-ribose) polymerase (PARP) using Western blotting with densitometry (lower panel). GAPDH was used as loading control. ns—not significant. Original images can be found in Figure S7.

As expected, the combined treatment of wild-type cells with CsA and CisPt markedly reduces cell viability and increases caspase 3/7 activity compared to the single treatment with CisPt alone (Figure 7A,B). More importantly, this combined treatment of wild-type cells yields the same effect as the single CisPt treatment in RIPK4 knockout cells. The observed changes are accompanied by a decrease in cyclin D1 levels and an increase in p53 and cleaved PARP levels in treated cells; however, this effect is slightly stronger in cells with RIPK4 deletion than in those treated with CsA and CisPt (Figure 7C). Poly (ADP-ribose) polymerase (PARP) cleavage is recognised as one of the hallmarks of apoptosis and caspase activation. On the other hand, A375^RIPK4.KO^ and WM266.4^RIPK4.KO^ cells, when additionally pretreated with CsA, showed an increased sensitivity to CisPt, at a lower concentration (10 µM), which was not seen at a concentration of 20 µM, which induced a strong toxic effect (Figure 8).

### 3.4. RIPK4 Downregulation as Well as Cyclosporin A Treatment Enhanced Sensitivity of Melanoma Cells to Doxorubicin

**Figure 9:**
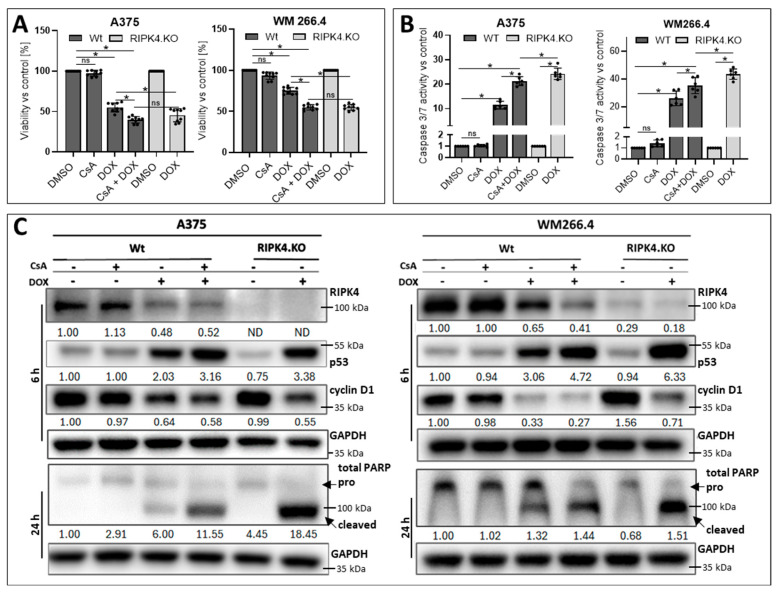
RIPK4 knockout has a similar effect on DOX-induced melanoma cell apoptosis as pretreatment with CsA. A375 ^RIPK4.KO^, WM266.4^RIPK4.KO^, and their wild-type (Wt) controls were pre-incubated for 1 h with CsA (5 μM) and then treated with DOX (4 μM), or DMSO, as a control, for 6–24 h. (**A**) Cell viability 24 h after treatment using MTT assay, *n* = 3; (**B**) caspase 3/7 activity, *n* = 3. Each bar represents the mean of at least three biological replicates ± SD. * *p* < 0.05 were considered significant. (**C**) Protein levels of RIPK4, p53, cyclinD1, and total Poly(ADP-ribose) polymerase (PARP) after indicated time periods using Western blotting with densitometry. GAPDH was used as loading control. ns—not significant. Original images can be found in Figure S8.

DOX may exhibit reduced efficacy due to the presence of MRPs, particularly ABCG2. Therefore, we further investigated whether ABCG2 downregulation, modulated by the absence of RIPK4, enhances the sensitivity of melanoma cells to doxorubicin. We aimed to determine if the observed effect would be comparable to CsA treatment. As shown in Figure 9A, DOX (4 µM) reduces the viability of A375^RIPK4.KO^ melanoma cells by approximately 46%, a level comparable to that observed in DOX/CsA-treated A375-Wt cells. Likewise, the viability of DOX/CsA-treated WM266.4 and WM266.4^RIPK4.KO^ cells decrease to 55%. However, DOX increases caspase 3/7 activity and cleaved PARP more efficiently in A375^RIPK4.KO^ and WM266.4^RIPK4.KO^ than in the dual treatment of DOX with CsA in wild-type cells (Figure 9B,C).

### 3.5. RIPK4 Overexpression in A375-dCas9 Cells Reduces the Action of CisPt and DOX

**Figure 10:**
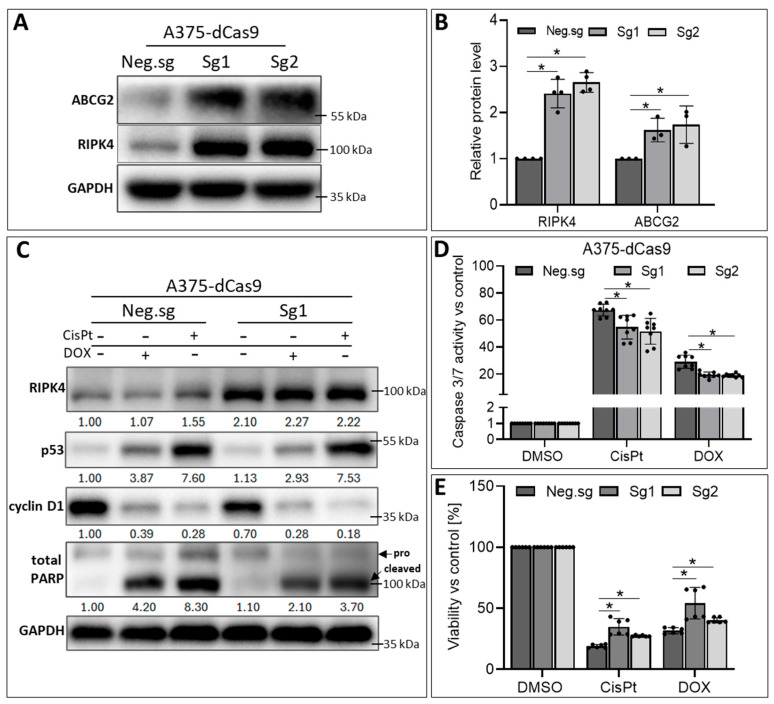
RIPK4 overexpression in A375-dCas9 cells attenuates sensitivity to CisPt and DOX. A375-dCas9 cells were transfected with two single sgRNAs targeting RIPK4 (RIPK4.sg1, RIPK4.sg2) or no targeting control (neg.sg). After 72 h of transfection, cells were incubated with CisPt (20 μM) or DOX (4 μM), or DMSO, as a control, for 24 h. (**A**,**B**) The expression levels of RIPK4 and ABCG2 were analysed using Western blotting along with densitometry, *n* = 3. GAPDH was used as a loading control. (**C**) The expression levels of RIPK4, p53, cyclin D1, and total Poly (ADP-ribose) polymerase (PARP) were analysed using Western blotting. GAPDH was used as a loading control. (**D**) Caspase3/7 activity assay after 24 h. *n* = 3 in duplicate. (**E**) Cell viability using MTT assay, *n* = 3. Each bar represents the mean ± SD of two biological replicates in triplicates. * *p* < 0.05 were considered significant. Original images can be found in Figure S9.

To confirm that RIPK4, through the regulation of ABCG2 expression, is crucial for the sensitivity of melanoma cells to CisPt and DOX treatment, we conducted RIPK4 overexpression in A375 cells. The results demonstrated a significant increase in ABCG2 protein levels upon RIPK4 overexpression (Figure 10A,B).

Additionally, cells with overexpression of RIPK4 and treated with DOX or CisPt showed an attenuated induction of p53 expression and were cleaved of the PARP protein compared to control cells (Figure 10C). These data suggest that cells are less sensitive to apoptosis induced by the selected drugs, as confirmed by a lower activation of caspase 3/7 activity (Figure 10D) and slightly higher viability as assessed using the MTT assay (Figure 10E).

## 4. Discussion

Chemotherapeutic resistance remains a challenge in improving the prognosis of patients with melanoma. Although DOX is one of the commonly used agents in cancer treatment [37,38,39], most melanoma patients are not sensitive to this drug, with a response rate that does not exceed 15% [39]. To prevent the development of cell resistance, multi-targeted therapy and the mechanisms leading to resistance are actively under investigation.

Cisplatin and doxorubicin provide a platform to study resistance mechanisms shared with other therapies. For example, resistance to cisplatin involves enhanced DNA repair, drug efflux, and apoptosis evasion, which are mechanisms that overlap with resistance to other drugs used in melanoma treatment. This makes these agents highly relevant for preclinical studies, even if they are not first-line treatments [40]. Additionally, studying these agents helps to elucidate the pathways involved in melanoma chemoresistance, potentially opening avenues for combination strategies or novel therapeutic targets that can complement existing treatments. Reversible electroporation, for example, allows for the effective delivery of cisplatin and bleomycin in electrochemotherapy [41].

We directed our attention to the kinase RIPK4, which has been shown to enhance sensitivity towards CisPt treatment of Tca-8113 cells in squamous cell carcinoma of the tongue [32]. Our previous studies confirmed that RIPK4 is heterogeneously expressed in melanoma specimens and cell lines [24,25]. Variable levels of RIPK4 in melanoma cells, ranging from very high to almost negligible [24,25], may reflect different sensitivities to CisPt. Cisplatin has shown only moderate efficacy in BRAF-mutated melanoma, mainly due to resistance mechanisms driven by the MAPK/ERK pathway, which is constitutively activated in melanoma with BRAFV600E mutation. This pathway promotes DNA repair and survival, thereby reducing the efficacy of cisplatin [42,43]. Our previous work [30] showed that BRAF inhibitors reduce RIPK4 expression, regardless of BRAF mutation status. However, we observed that the effect of these inhibitors on the ERK pathway varied depending on the mutation, suggesting that RIPK4 may regulate alternative signalling pathways in different genetic contexts. We found that cells with the lowest levels (BLM) and highest levels (WM266.4) of RIPK4 were the most resistant to CisPt. Such a result suggests that not only RIPK4 but also other features, such as BRAF mutation status, may influence the sensitivity of melanoma cells to cisplatin. Interestingly, BLM cells, which are BRAF wild-type and NRAS mutant, are more resistant to cisplatin compared to WM266.4 and the other BRAF mutant cell lines tested. The question of whether RIPK4 levels affect cisplatin sensitivity more in melanoma cells with BRAF mutations than in those with NRAS or BRAF wild-type mutations warrants further investigation in future studies.

To investigate the precise mechanism of action of RIPK4, we performed gene silencing in two ways: using siRNA and through stable knockout using CRISPR/Cas9. Both silencing and knockout of RIPK4 did not induce apoptosis in A375 or WM266.4 cells. However, it sensitised melanoma cells to CisPt and DOX, leading to the inhibition of cell proliferation and increased apoptosis.

So far, there have been no studies that explain the mechanism of RIPK4 involvement in the resistance of cells to CisPt in melanoma. Considering that metallothioneins (MTs) are responsible for protection against heavy metal toxicity and, consequently, in chemoresistance [44,45], we investigated whether RIPK4 could be involved in the regulation of the expression of these proteins. Our results indicate an increase in MTX1 expression in WM266.4^RIPK4.KO^ cells, suggesting a potential link between RIPK4 and MTX1 in the regulation of CisPt susceptibility. However, the opposite effect was observed in A375^RIPK4.KO^ cells, where MT1X expression decreases. These results indicate that RIPK4 may impact MT1X expression but that it is not the main pathway responsible for RIPK4-dependent CisPt resistance in melanoma cells. It should be noted that overexpression of metallothionein has been shown to be a prognostic factor for the progression and survival of melanoma [46].

Resistant to apoptosis despite DNA damage or pro-apoptotic signals, melanoma cells pose a challenge in understanding the mechanisms behind their evasion of programmed cell death. This resistance may be attributed to inactivating mutations in suppressor genes, as well as genes encoding caspases and pro- and anti-apoptotic proteins. Many data indicate an imbalance between pro- and anti-apoptotic factors in cancer cells, including melanoma [47]. Our previous observations on WM266.4 cells with transient silencing of RIPK4 did not indicate changes in the activity of BAD and BCL-2 genes, but they did reveal a potential link between BIRC3 and RIPK4 in the regulation of IL6 and IL8 expression [30,31]. Therefore, in our second approach, we focused on BIRC5 protein belonging to the IAP family, which plays a key role in avoiding apoptosis by blocking apoptosis signalling pathways and promoting survival. Members of the IAP family are frequently overexpressed in tumours, contributing to cancer cell survival, chemotherapy resistance, disease progression, and poor prognosis [48]. In fact, we did not find that RIPK4 knockout affects BIRC5 expression, but we confirmed that it downregulates BIRC3 in both tested melanoma lines. Bertrand et al. indicated a direct interaction between RIPK4 and BIRC3 (known as cIAP2) [49].

Our previous studies have shown that downregulation of RIPK4 impairs signalling pathways important for the survival and progression of melanoma, such as NFκB [24,31] and Wnt/β-catenin [25], which are also involved in the regulation of ABCG2 protein levels [50,51]. In the last attempt, we focused on MPRs, such as ABCB1, ABCC1, and ABCG2. We found that RIPK4 plays a role in the regulation of ABCG2 levels, as demonstrated by a decrease in ABCG2 expression after RIPK4 knockout and an increase with overexpression. As multiple studies have shown, increased expression of ABCG2 results in resistance to anticancer drugs [52,53]. In fact, we found that overexpression of RIPK4 in A375-dCas9 cells reduces resistance to CisPt-induced apoptosis, as evidenced by lower caspase 3/7 activation and attenuated p53 induction and PARP-cleaved protein expression compared to control cells. Furthermore, we confirm that the CisPt treatment of RIPK4.KO cells reduces the activity of AKT and the Wnt/β-catenin pathway more efficiently than either of these agents, indicating the association of these pathways with RIPK4 and CisPt.

ABCG2 is the main protein transporter responsible for the development of DOX resistance [54]. Upon entering the cell, DOX binds to the proteasome, forming a complex that translocates to the nucleus, where DOX dissociates and binds to DNA, blocking the cleavage activity of topoisomerase II and interfering with nucleic acid synthesis [55,56,57,58]. DNA damage leads to cell cycle arrest in the G0/G1 and G2/M phases and programmed cell death [59]. Similar to CisPt, DOX can also bind to mtDNA [60]. The expression of ABCG2 has been observed in various tumours, including melanoma, suggesting that ABCG2 represents a common mechanism of clinical drug resistance [61,62]. Clinical studies indicate that high expression of ABCG2 in tumours is a prognostic marker for poor clinical outcomes [63]. Therefore, we investigated CRISPR/Cas9-mediated RIPK4 downregulation, as well as overexpression on apoptosis induction by DOX. The results confirmed the engagement of RIPK4 in DOX-induced apoptosis. Additionally, treatment of wild-type melanoma cells simultaneously with CsA and CisPt or CsA and DOX increases caspase 3/7 activity and reduces the survival of melanoma cells compared to RIPK4.KO cells, regardless of the melanoma cell lines studied. These observed changes were accompanied by a decrease in cyclin D1 levels and an increase in p53 and cleaved PARP levels in CsA-treated cells.

Although cyclosporine A is not a highly selective inhibitor of ABCG2, but rather a broad-spectrum modulator of multidrug resistance [64], it demonstrates effective inhibitory properties against ABCG2 protein [65]. In our model system, the ABCG2 protein level is significantly decreased in RIPK4 knockout cells (65–70%) and the effect of RIPK4 knockout on sensitivity to CisPt is comparable to that of Wt cells incubated with CsA. In cells with knockout, the level of other proteins from the MDR family, including the Pgp protein, does not change. Similar results were obtained in experiments using DOX, which is a typical BCRP substrate used to stimulate the expression of this protein in cells. An additional advantage of CsA is its extensive clinical use providing well-established data on safety (half-life, adverse effects, toxicity, etc.). Therefore, it is a good candidate to choose from among a number of other MDR inhibitors [66]. However, because both CsA and DOX interact with Pgp, the involvement of this protein in the observed final effect cannot be excluded.

Our study provides preliminary evidence of crosstalk between RIPK4, BIRC3, and ABCG2. Uncovering the mechanisms underlying this interaction could potentially pave the way for developing more effective therapies. While our findings demonstrate concurrent alterations in RIPK4, ABCG2, and BIRC3, as well as changes in apoptosis rates, the precise mechanisms by which ABCG2 and BIRC3 mediate the effects of RIPK4 on the efficacy of selected chemotherapeutics remain to be fully characterised. The results of these studies suggest the involvement of RIPK4 in the observed resistance to CisPt or DOX. However, whether downregulation of RIPK4 can serve as a promising therapeutic strategy to overcome resistance to CisPt or DOX in mul-targeted melanoma chemotherapy requires further investigation on a broader panel of melanoma cell lines and preclinical models.

## 5. Conclusions

In conclusion, this is the first demonstration that RIPK4 plays a role in the regulation of ABCG2 levels, as demonstrated by a decrease in ABCG2 expression after RIPK4 knockout and an increase with overexpression. Furthermore, we confirm that RIPK4 deletion enhances the effects of CisPt or DOX similarly to preincubation with cyclosporin A, an ABCG2 inhibitor. The results of these studies suggest the involvement of RIPK4 in the observed resistance to CisPt or DOX.

## Data Availability

Data are contained within the article and Supplementary Materials.

## Supplementary Materials

The following supporting information can be downloaded at: https://www.mdpi.com/article/10.3390/biom14121573/s1, Figures S1–S9: Contain the original blots associated with the figures containing the Western blot results. Table S1. List of general reagents and their sources; Table S2. List of TaqMan probes used and purchased from Thermo Fisher Scientific/Invitrogen; Table S3. Antibodies used for Western blot (WB) analysis.

## Abbreviations

AnxV	Annexin V
ABCB1	ATP-binding cassette super-family B member 1 (P-glycoprotein)
ABCC1	ATP-binding cassette super-family C member 1 (MRP1)
ABCG2	ATP-binding cassette super-family G member 2 (BCRP)
BIRC3	Baculoviral IAP repeat containing 3 (c-IAP2)
BIRC5	Baculoviral IAP repeat containing 5
CisPt	Cisplatin
CsA	Cyclosporine A
DOX	Doxorubicin
IAP	Inhibitors of apoptosis proteins
MCL1	Myeloid cell leukemia-1
MPR	Multidrug resistance proteins
MT1X	Metallothionein 1X
7AAD	7-amino-actinomycin D
PARP	Poly (ADP-ribose) polymerase
RIPK4	Receptor-interacting protein kinase
