# Supplementary material for: RIPK4 Downregulation Reduces ABCG2 Expression, Increasing BRAF-Mutated Melanoma Cell Susceptibility to Cisplatin- and Doxorubicin-Induced Apoptosis
Source: Biomolecules. 2024 Dec 10;14(12):1573. doi: 10.3390/biom14121573 (PMC11674099; doi:10.3390/biom14121573)
Supplement: Supplementary file 1 [file biomolecules-14-01573-s001.zip › biomolecules-3304253-supplementary.pdf]

biomolecules

MDPI

Supplementary Materials

RIPK4 Downregulation Reduces ABCG2 Expression, Increasing BRAF-Mutated Melanoma Cell Susceptibility to Cisplatin- and Doxorubicin-Induced Apoptosis

Bartlomiej Olajossy [1,2], Norbert Wronski [1,2], Ewelina Madej [1], Joanna Komperda [1], Malgorzata Szczygiel [1], Agnieszka Wolnicka-Glubisz [1,*]

1 Department of Biophysics and Cancer Biology, Faculty of Biochemistry, Biophysics and Biotechnology, Jagiellonian University, Gronostajowa Street 7, 30-387 Krakow, Poland; bartek.olajossy@doctoral.uj.edu.pl (B.O.); norbert.wronski@doctoral.uj.edu.pl (N.W.); em939@cam.ac.uk (E.M.); gosia.szczygiel@uj.edu.pl (M.S.)
2 Doctoral School of Exact and Natural Sciences, Jagiellonian University, 30-387 Krakow, Poland
* Correspondence: a.wolnicka-glubisz@uj.edu.pl; Tel.: +48-12-664-65-26

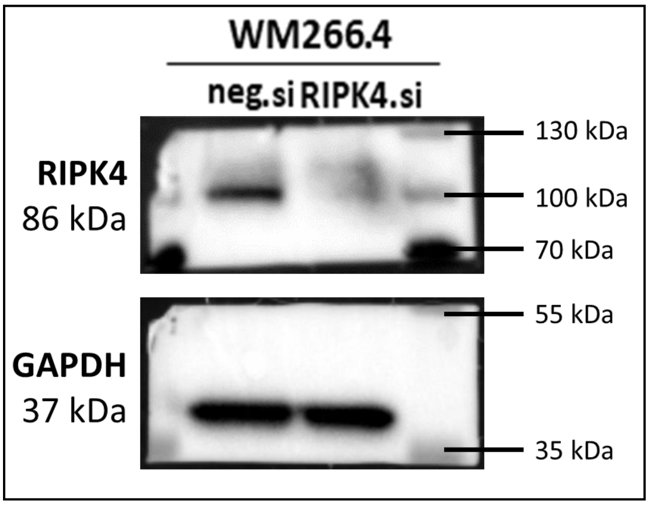

Figure S1. Original unedited blot related to figure 1B. The protein levels of RIPK4 and GAPDH by Western blot. Membrane was cut before hybridization with antibodies.

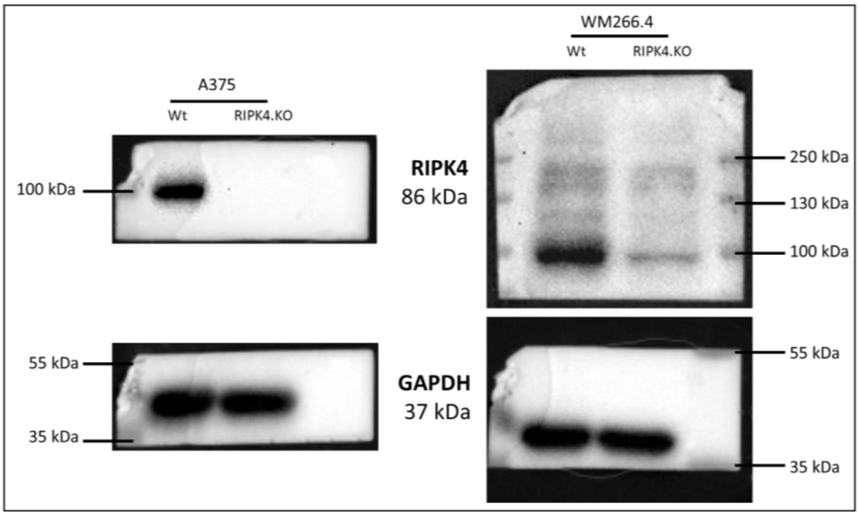

Figure S2. Original unedited blot related to figure 2A (The level of RIPK4 in A375$^{RIPK4.KO}$, WM266.4 $^{RIPK4.KO}$cells and their parental lines (wild-type, Wt). Membrane was cut before hybridization with antibodies.

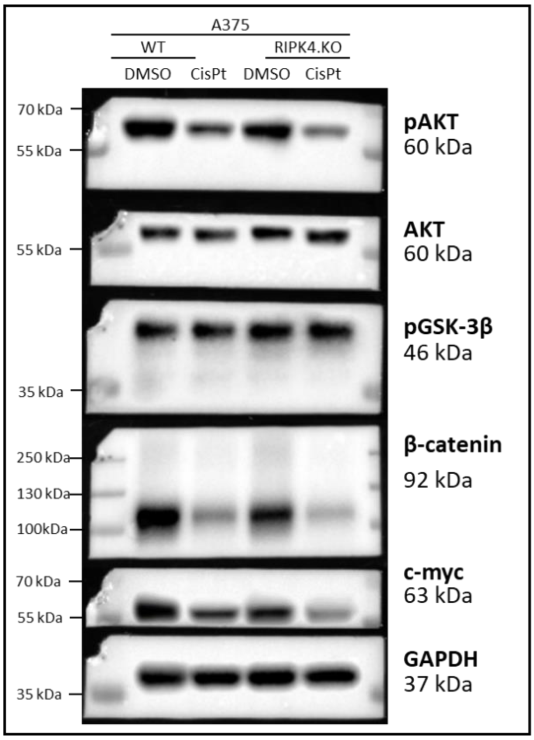

Figure S3 Original unedited blot related to figure 3. The protein levels of pAKT, AKT, pGSK3β, β-catenin, c-myc, GAPDH in knockout cells (RIPK4.KO) and their parental lines (Wt) were assessed using Western blotting. Membrane was cut before hybridization with antibodies.

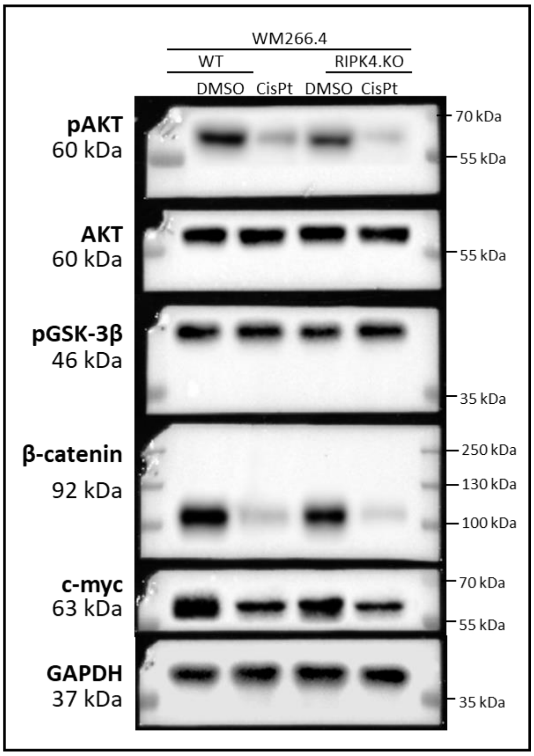

Figure S4 Original unedited blot related to figure 4. The protein levels of pAKT, AKT, pGSK3β, β-catenin, c-myc in knockout cells (RIPK4.KO) and their parental lines (Wt) were assessed using Western blotting. Membrane was cut before hybridization with antibodies.

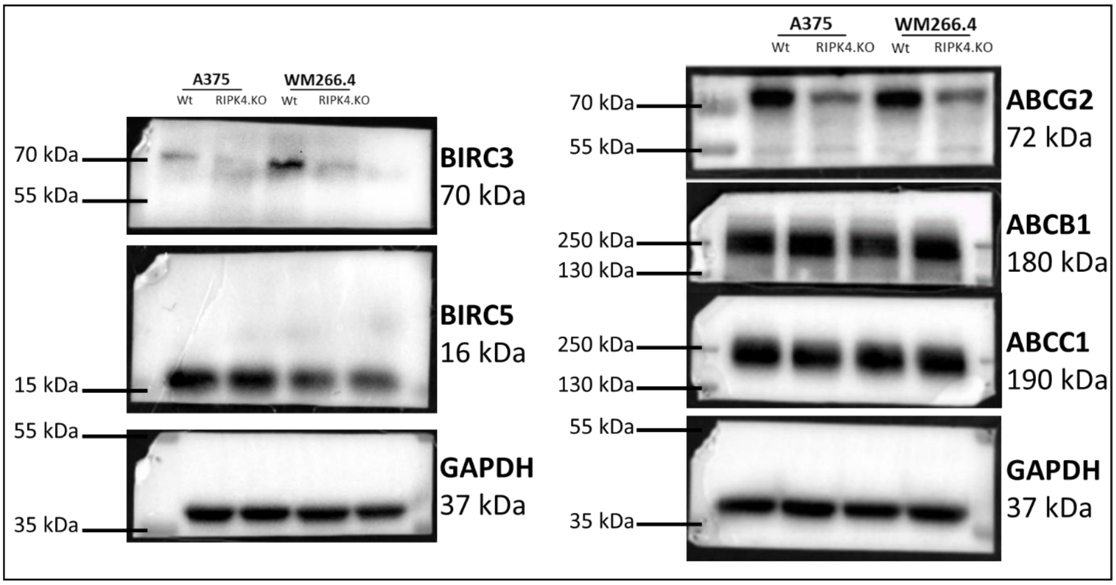

Figure S5. Original unedited blot related to figure 6. The protein levels of BIRC5, BIRC3, ABCB1, ABCC1, ABCG2, GAPDH in knockout cells (RIPK4.KO) and their parental lines (Wt) were assessed using Western blot. Membrane was cut before hybridization with antibodies.

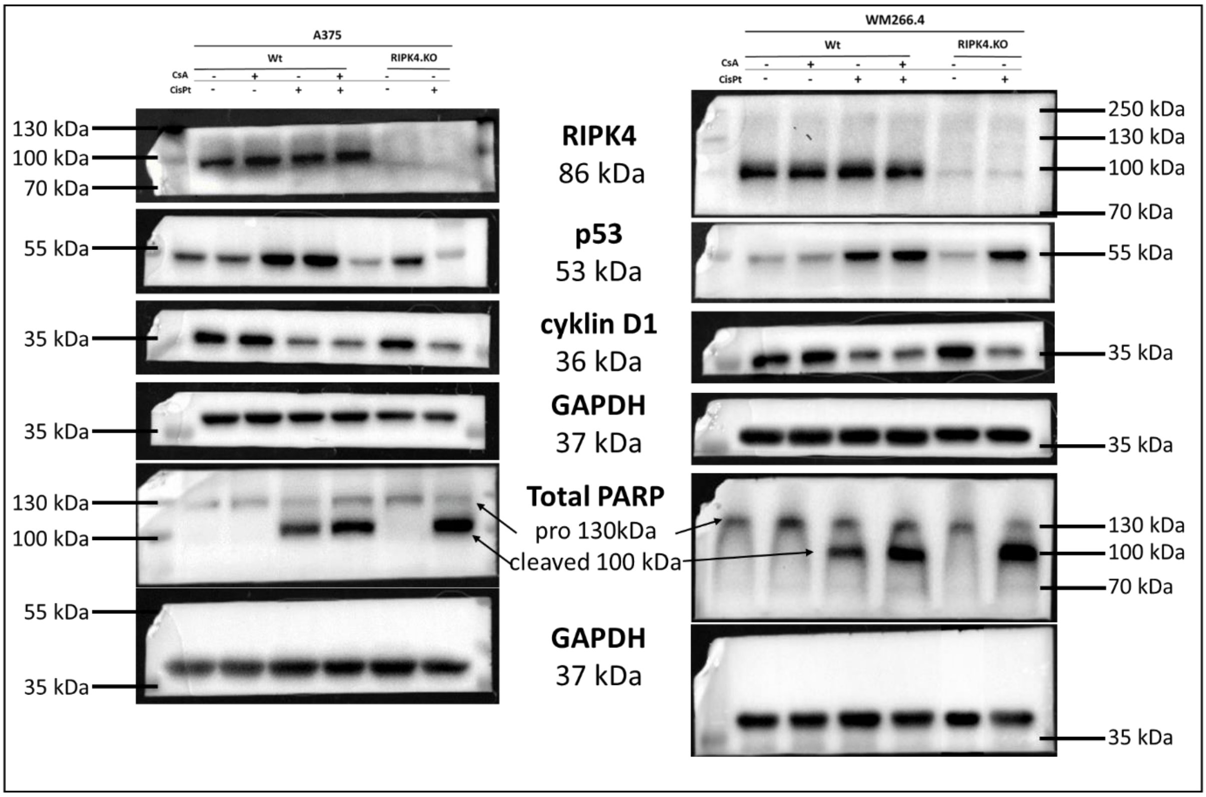

Figure S6. Original unedited blot related to figure 7C. Protein levels of RIPK4, p53, cyclinD1, and total Poly(ADP-ribose) polymerase (PARP), GAPDH by Western blot. Membrane was cut before hybridization with antibodies.

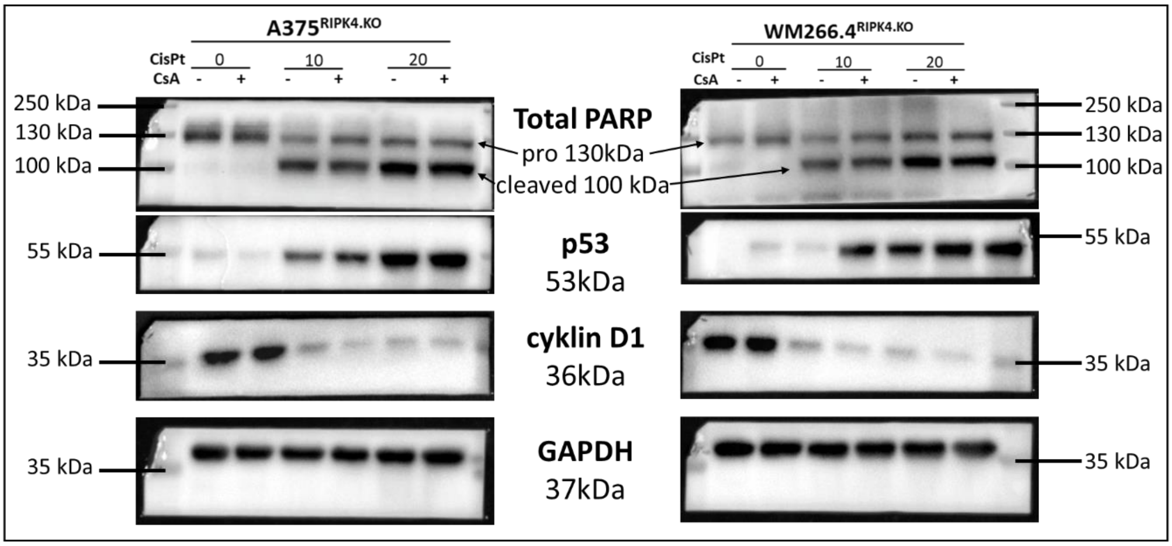

Figure S7. Original unedited blot related to figure 8 A and B. Protein levels of p53, cyclin D1, and total Poly(ADP-ribose) polymerase (PARP), GAPDH by Western blot. Membrane was cut before hybridization with antibodies.

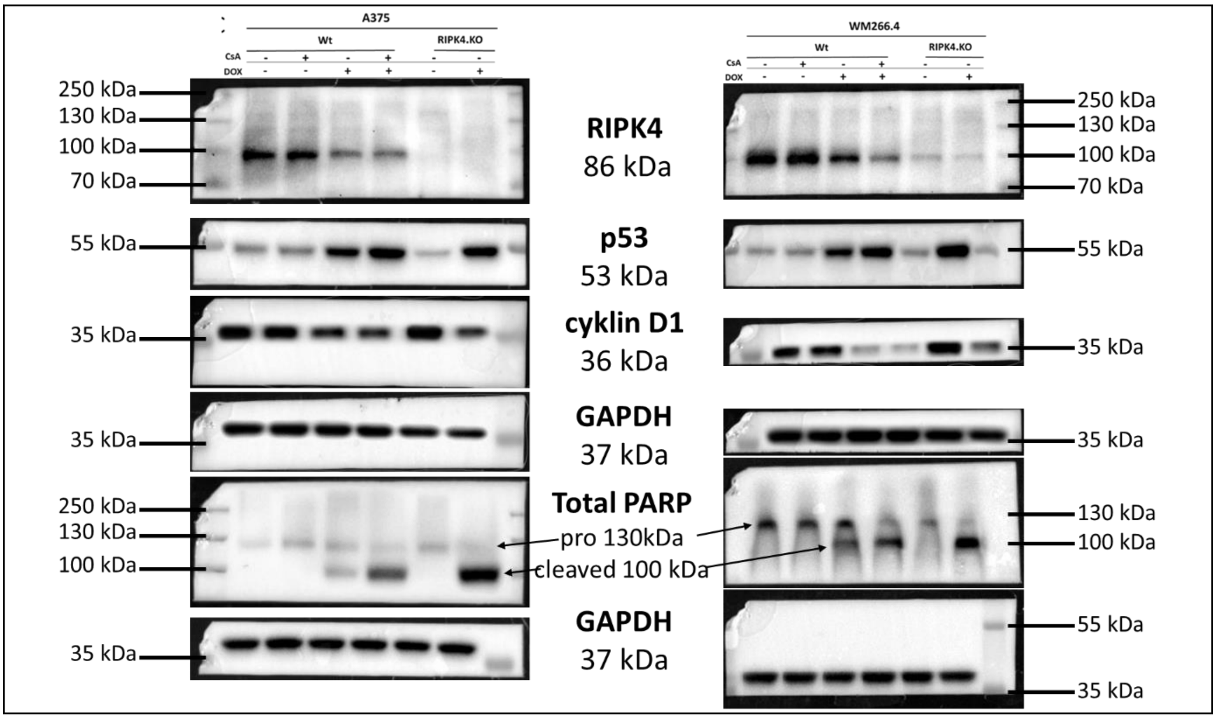

Figure S8. Original unedited blot related to figure 9C. Protein levels of RIPK4, p53, cyclin D1, and total Poly(ADP-ribose) polymerase (PARP), GAPDH by Western blot. Membrane was cut before hybridization with antibodies.

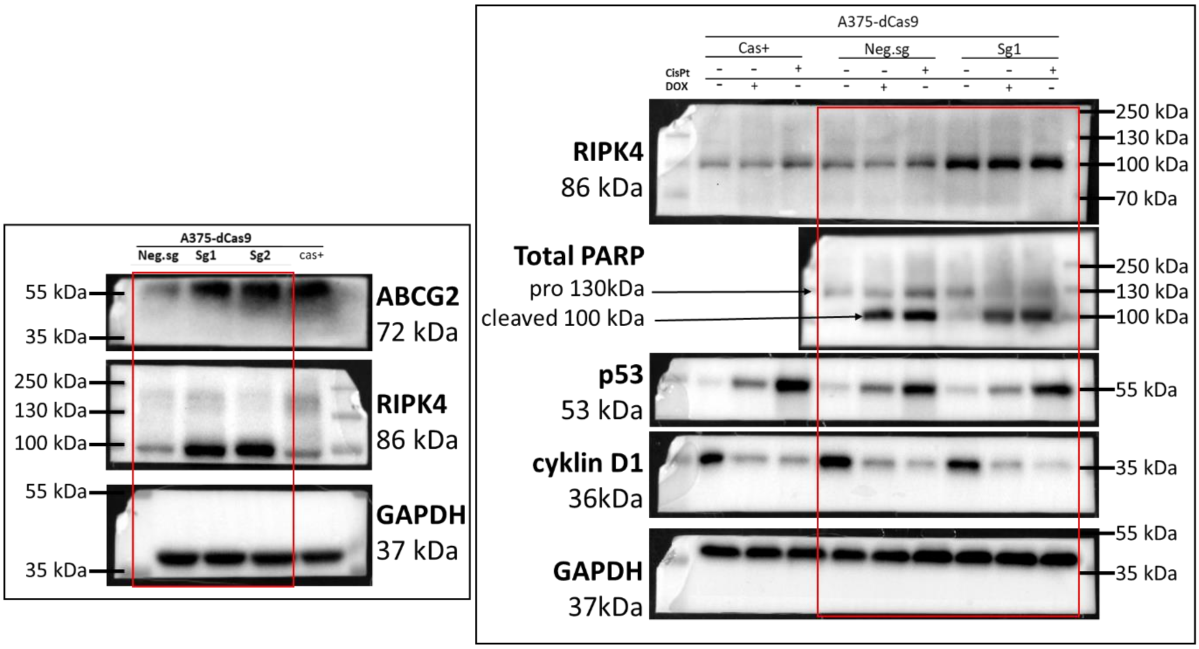

Figure S9. Original unedited blot related to figure 10 A and C. (left) The expression levels of RIPK4, ABCG2, GAPDH were analysed using Western blotting. (right) The expression levels of RIPK4, p53, cyclin D1, and total Poly (ADP-ribose) polymerase (PARP), GAPDH were analysed using Western blotting.

Reagent	Cat. No	manufacturer	country
cyclospoorin	C3662	Sigma-Aldrich	St. Louis, USA
cisplatin	232120		
doxorubicin	D1515		
Thiazolyl blue tetrazolium bromide	M5655		
protease inhibitor cocktail	P8340		
PhosSTOP phosphatase inhibitor cocktail	4906845001		
Polybrene	TR1003		
RIPA buffer	R0278		
Bicinchoninic Acid Protein Assay Kit	B9643		
penicillin 150 U/mL, and streptomycin 100 g/mL	P4333		
The 12% Bis-Tris gels—TGX™ FastCast™ Acrylamide Kit	1610175	Bio-Rad	Hercules, USA
ClarityWestern ECL substrate	1705060		
PVDF membranes 0.2 _m pore size	ISEQ00010	Milipore	Burlington, USA
AnnexinV-FITC early apoptosis detection kit	6592S	Thermo Fisher Scientific	Waltham, USA
7AAD	00-6993-50		
Lipofectamine 2000	11668-030		
RIPK4-specific Silencer® Select siRNAs ID: s28865	4390824		
Silencer® Select Negative Control No. 2	4390846		
sgRNAs	PQ-005308-01-0002	Horizon Discovery	Waterbeach, UK
non-targeting control crRNA: tracrRNA	U-009500-01-05		
DharmaFECT 4 Transfection Reagent	T-2001-02		
Bovine serum	ALB001	BioShop	Burlington, Canada
Fetal bovine serum	10500064	Gibco	New Yor, USA
Caspase 3/7 kit	G8091	Promega	Madison, USA
MycoAlert PLUS Mycoplasma Detection Kit	LT07-118	Lonza Bend	Bend, USA

RPMI1640	01-100-1A	BI/ Sartorius	Getynga, Germany
Methanol	621990110	POCH	Gliwice, Poland
Formaldehyde	432173111		
Dimethyl sulfoxide	363550117		
Puromycin	Ant-pr-1	InvivoGen	San Diego, USA
Total RNA Mini Plus	036	A&A Biotech-nology	Gdańsk, Po-land

Table S1. List of general reagents and their sourses

Gene	ID:
BIRC3	Hs00985030_g1
MCL1	Hs03043899_m1
MT1X	Hs00745167_sH
GAPDH	Hs02786624_g1

Table S2. List of TaqMan probes used and purchased from Thermo Fisher Scientific / Invitrogen.

Antibody	Source	Dilution	Cat. No.	Company
anti-RIPK4	rabbit	1:2000	12636	Cell Signaling Technology
anti-GAPDH			5174	
anti-BIRC5			2808	
anti-BIRC3 (c-IAP2)			3130	
anti-PARP			9542	
anti-ABCG2			42078	
anti-phospho-AKT (Ser 473)			4060	
anti-phospho-GSK-3β (Ser9)			9323	
anti-C-myc			5605	
anti- β-catenin	mouse	1:4000	sc-59737	Santa Cruz Biotechnology
anti-p53			sc-126	
anti-ABCC1	rat	1:50	ALX-801-007	Enzo
anti-ABCB1	mouse	1:100	ALX-801-002	
HRP-conjugated goat anti-mouse	goat	1:4000	554002	BD Pharmingen

HRP-conjugated goat anti-rabbit		1:2000	7074	Cell Signaling Technology
HRP-conjugated rabbit anti-rat	rabbit	1:100	P0450	Dako

Table S3. Antibodies used for Western Blot (WB) analysis
